# Tyrosine 23 Phosphorylation of Annexin A2 Promotes Proliferation, Invasion, and Stat3 Phosphorylation in the Nucleus of Human Breast Cancer SK-BR-3 Cells

**DOI:** 10.7497/j.issn.2095-3941.2012.04.005

**Published:** 2012-12

**Authors:** Yu-qing Wang, Fei Zhang, Ran Tian, Wei Ji, Yan Zhou, Xiu-mei Sun, Yuan Liu, Zhi-yong Wang, Rui-fang Niu

**Affiliations:** Tianjin Medical University Cancer Institute and Hospital; Key Laboratory of Cancer Prevention and Therapy, Tianjin; Key Laboratory of Breast Cancer Prevention and Therapy, Ministry of Education, Tianjin 300060, China

**Keywords:** Annexin A2, tyrosine, mutation, phosphorylation, Stat3 transcription factor

## Abstract

**Objective:**

To investigate the role of tyrosine 23 (Tyr23) phosphorylation of Annexin A2 (Anxa2) in regulating the proliferation and invasion of human breast cancer SK-BR-3 cells.

**Methods:**

A panel of lentivirus plasmids expressing Anxa2-wide type (Ana2-WT), Anxa2-Y23A, and Anxa2-Y23D was generated and infected with SK-BR-3 cells. The monoclonal strains were screened. The expression of Anxa2-WT, Anxa2-Y23A, and Anxa2-Y23D was determined by Western blot analysis. The ability of the cells to proliferate was detected through an MTT [3-(4,5-Dimethylthiazol-2-yl)-2,5-diphenyltetrazolium bromide] test. Boyden chamber assays were employed to examine migration and invasion abilities. The interaction between Anxa2 and Stat3 was analyzed by immunoprecipitation analyses. Nucleoprotein and cytosolic protein were extracted from SK-BR-3, Anxa2-WT, Anxa2-Y23A, and Anxa2-Y23D cells to analyze the expression and localization of Stat3 phosphorylation.

**Results:**

The monoclonal strains constitutively expressing Anxa2-WT, Anxa2-Y23A, and Anxa2-Y23D were screened. Both Anxa2-WT and Anxa2-Y23D enhanced the proliferation, migration and invasion abilities of SK-BR-3 cells (*P*<0.05). Immunoprecipitation analysis revealed that Anxa2 and Stat3 interacted with each other, and the expression of Stat3 phosphorylation in the nucleus was enhanced by Anxa2-Y23D.

**Conclusions:**

Tyr23 phosphorylation of Anxa2 promotes the proliferation and invasion of human breast cancer SK-BR-3 cells and the phosphorylation of Stat3 in the nucleus.

## Introduction

According to the global cancer statistics, breast cancer is the most frequently diagnosed cancer and the leading cause of cancer death among females, accounting for 23% of the total cancer cases and 14% of the cancer deaths^[^^1^^]^. In China the morbidity of breast cancer rises year by year, and diagnosis is usually made at an early age. High metastatic potential and drug resistance account for the failure of breast cancer treatments. Annexin A2 (Anxa2), a member of the Annexin family of calcium-dependent phospholipid binding proteins^[^^2^^]^, was reported to be abundantly expressed in many cancer tissues and is believed to play an important role in tumorigenesis and breast cancer progression^[^^3^^]^. Structurally, Anxa2 consists of a highly conserved C-terminal core domain and a variable N-terminal domain with p11 proteins and t-pA binding sites and phosphorylation sites for different kinases, for example, Ser25 for protein kinase C (PKC) phosphorylation and tyrosine 23 (Tyr23) for Src kinase phosphorylation^[^^3^^,^^4^^]^. Tyr23 phosphorylation of Anxa2 mediates cell scattering and branching morphogenesis^[^^4^^]^, and regulates Rho-mediated actin rearrangement and cell adhesion. The dynamic remodeling of the actin cytoskeleton is required for cell spreading, motility, and migration^[^^5^^]^. A more recent study demonstrated that Tyr23 phosphorylation-dependent cell-surface localization of Anxa2 is required for the invasion and metastases of pancreatic cancer^[^^4^^]^. However, little is known about the effects of Tyr23 phosphorylation of Anxa2 on the proliferation and invasion abilities of breast cancer cells and on the localization of the phosphorylation signal transducer and activator of transcription (Stat3). In the present study, Tyr23 was mutated to an alanine residue, creating a non-phosphorylatable mutant or an aspartic acid mimicking constitutive phosphorylation. A panel of experiments was performed on the two mutants, Anxa2-Y23A and Anxa2-Y23D, as well as on Anxa2-WT cells to detect the probable function of Tyr23 phosphorylation in regulating the migration and metastases of SK-BR-3 cells and the localization of Stat3 phospohrylation in SK-BR-3 cells.

## Materials and Methods

### Reagents

Rabbit monoclonal and mouse monoclonal anti-Annexin A2, mouse monoclonal anti-phospho-Annexin A2, and anti-actin and anti-GFP antibodies were bought from Santa Cruz (Santa Cruz Biotechnology, CA, USA). Rabbit monoclonal anti-Stat3 antibodies and anti-Phospho-Stat3 antibodies were purchased from Cell Signaling Technology (Danvers, MA, USA). Rabbit monoclonal and mouse monoclonal anti-IgG as well as the secondary antibodies, goat anti-rabbit or anti-mouse IgG or donkey anti-goat IgG conjugated to fluorescein isothiocyanate (FITC) or Texas Red, were obtained from Santa Cruz. Lipofectamine 2000 was purchased from Invitrogen (Carlsbad, CA, USA). All other chemicals were purchased from Sigma (St. Louis, MO, USA).

### Construction of Anxa2 expression vector and site-directed mutagenesis

The full-length human Anxa2 cDNA was amplified through reverse transcription polymerase chain reaction (RT-PCR). The PCR products (1033bp) were cloned in the Xho I and Bam HI sites of pEGFP-N3 vector. pEGFP-N3-Anxa2^-WT^ was referred to as Anxa2-WT in this study. The mutants of pEGFP-N3-Anxa2^-Y23A^ and pEGFP-N3-Anxa2^-Y23D^ from this cDNA, in which Tyr23 was replaced by alanine and aspartic acid, were cloned by sequential site-directed mutagenesis reactions using Quik-Change site-directed mutagenesis (Stratagene) according to the manufacturer’s instructions. The recombinant plasmids were confirmed by a testing sequence, and the quality of the recombinant protein was checked with a fluorescence microscope and sodium dodecyl sulfate polyacrylamide gel electrophoresis (SDS-PAGE).

### Cell treatment conditions and screening stable transfectants

Breast cancer SK-BR-3 cells were cultured as described previously^[^^6^^]^. The SK-BR-3 cells were infected with the lentivirus plasmids expressing Anxa2-WT, Anxa2-Y23A and Anxa2-Y23D or an empty vector using Lipofectamine 2000 according to the manufacturer’s procedures. Stable transfectants were selected with 800 µg/mL G418. Clones were picked and maintained in a complete medium containing 100 µg/mL G418. The expression of Anxa2-WT, Anxa2-Y23A, and Anxa2-Y23D was analyzed by Western blot with GFP and green fluorescence.

### Preparation of cytoplasmic and nuclear protein fractions

The cells were collected in a 1.5 cm Eppendorf tube and suspended by a fractionation buffer. The cells were passed through a 25 G needle 10 times using a 1 mL syringe. The cells were left on ice for 20 min. The lysate was then centrifuged at 800 *g* for 5 min. The supernatant was saved as a cytoplasmic fraction. The nuclear pellet was resuspended by a fractionation buffer again, passed through a 25 G needle several times, and centrifuged again at 800 *g* for 10 min. The buffer was removed, and the nuclear pellet was suspended in the nuclear buffer (standard lysis buffer with 10% glycerol and 0.1% SDS).

### Whole cell lysis, immunoprecipitation, and immunoblot analysis

Briefly, cells were placed on ice and incubated for 30 min in a lysis buffer (50 mM Tris at pH7.4, 10% glycerol, 0.1% SDS, 10 mM NaF, 2 mM PMSF, and 1 mM dithiothreitol). The lysed cells were then centrifuged at 12,000 *g* for 20 min at 4°C. The supernatants were used for immunoprecipitation and immunoblot analysis. The obtained supernatant was mixed with protein A/G beads (Invitrogen) at 4°C, incubated for 1 h, and centrifuged for 30 s at 10,000 *g*. The supernatant was then incubated overnight with Stat3/Anxa2 antibody and protein A/G beads and centrifuged for 30 s at 10,000 *g*. Proteins were prepared by whole-cell lysis and subcellular fractionation for immunoblot. A SDS-PAGE sample buffer was boiled for 5 min to 10 min. The proteins were then separated by electrophoresis on 12% SDS-PAGE gels. The proteins were transfected onto a polyvinylidene fluoride membrane, which was blocked with 5% skim milk and incubated overnight with primary antibodies at 4°C. After incubation in an Odyssey blocking buffer with secondary antibodies for 1 h at room temperature, the immunoreactive bands were then determined by image scanning on an Image Station LI-COR Odyssey imaging system. The bands were analyzed using the imageJ software.

### Immunofluorescence studies

Various cells were grown on 18 mm^2^ round glass coverslips (6×10^3^/coverslip). The cells were fixed with 4% paraformaldehyde at room temperature for 10 min labeling. After fixation, the cells were permeabilized with 0.2% Triton X-100 in PBS and quenched with 50 mM NH_4_Cl/PBS. Several washing steps later, the coverslips were blocked with 3% BSA (bovine serum albumin), then incubated in primary antibodies (1:100) and secondary antibodies (goat anti-rabbit or mouse IgG-FITC; 1:1000). The cells were incubated for 10 min with 1 µg/mL DAPI (4’, 6-diamidino-2-phenylindole) for cellular nuclei staining. Finally, the coverslips were mounted on slides with mowiol and observed under an Olympus BX40 epifluorescence confocal microscope. The images were captured with MagnaFire software and processed using Photoshop.

### Proliferation, migration and invasion assays

Proliferation, migration, and invasion assays were performed as described previously^[^^7^^]^.

### Statistical analysis

Student’s *t*-test was used to determine whether a significant difference exists between two means (*P*<0.05). In multiple means, the difference was determined by one-way analysis of variance (*P*<0.05). The significant differences are marked in the graphs. Triplicate experiments were done for all the samples.

## Results

### Lentivirus based plasmids, pCDH-EGFP-Anxa2^WT^, pCDH-EGFP-Anxa2^Y23A^, and pCDH-EGFP-Anxa2^Y23D^, were established successfully

**Figure 1:**
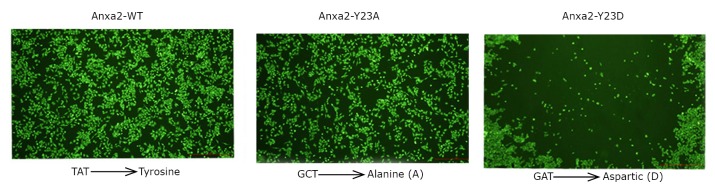
Results of the testing sequence for the three lentivirus plasmids. Lentivirus-based plasmids pCDH-EGFP-Anxa2^WT^, pCDH-EGFP-Anxa2^Y23A^, and pCDH-EGFP-Anxa2^Y23D^ were established successfully.

To test whether the phosphorylation of Anxa2 at Tyr23 is important for the proliferation and invasion of breast cancer SK-BR-3 cells, we generated a panel of plasmids pEGFP-N3-Anxa2^WT^, pEGFP-N3-Anxa2^Y23A^, and pEGFP-N3-Anxa2^Y23D^ (**Figure 1**). pEGFP-N3-Anxa2^WT^ and its mutants were digested by two restriction enzymes, and directionally inserted into pCDH. Thereupon, lentivirus based plasmids pCDH-EGFP-Anxa2^WT^, pCDH-EGFP-Anxa2^Y23A^, and pCDH-EGFP-Anxa2^Y23D^ were established successfully.

### Anxa2-WT and Anxa2-Y23D increased proliferation of SK-BR-3 cells

**Figure 2:**
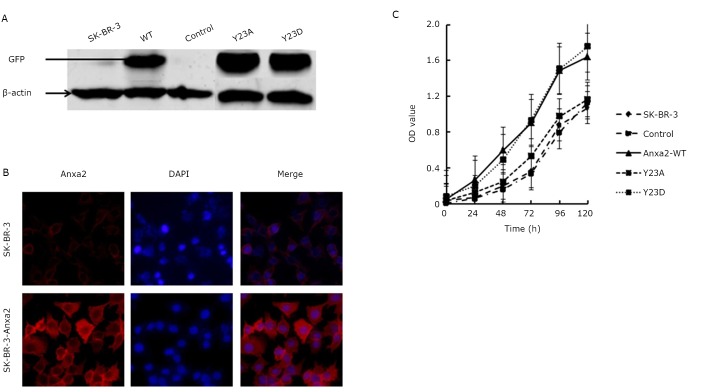
Anxa2-WT and Anxa2-Y23D enhanced the proliferation of SK-BR-3 cells. A: Western blot analysis of GFP in SK-BR-3, control, Anxa2-WT, Anxa2-Y23A and Anxa2-Y23D cells. B: Immunofluorescence study of Anxa2-WT. Anxa2 was evidently overexpressed in SK-BR-3-Anxa2 cells. C: Proliferation ability was markedly enhanced in Anxa2-WT and Anxa2-Y23D cells. Triplicate experiments were done for B and C. The statistical significance was assessed by one-way ANOVA.

Lentivirus plasmids expressing Anxa2-WT, Anxa2-Y23A, or Anxa2-Y23D were infected with SK-BR-3 cells, and the strains that constitutively expressing these proteins were screened. Western blots were stained for green fluorescent protein (GFP) to determine the exact expression of Anxa2-WT, Anxa2-Y23A, and Anxa2-Y23D proteins in the SK-BR-3 cells (**Figure 2A**). Immunofluorescence studies of Anxa2 also showed an overexpression of Anxa2 in the SK-BR-3 cells, as depicted in **Figure 2B**. MTT assay results showed that compared with SK-BR-3, control (SK-BR-3 cells expressing empty pCDH lentivirus), and Anxa2-Y23A cells, the Anxa2-WT and Anxa2-Y23D cells exhibited an enhanced ability of duplication (**Figure 2C**). Anxa2-WT and Anxa2-Y23D enhanced the proliferation of SK-BR-3 cells.

### Anxa2-WT and Anxa2-Y23D increased migration and invasion of SK-BR-3 cells *in vitro*

**Figure 3:**
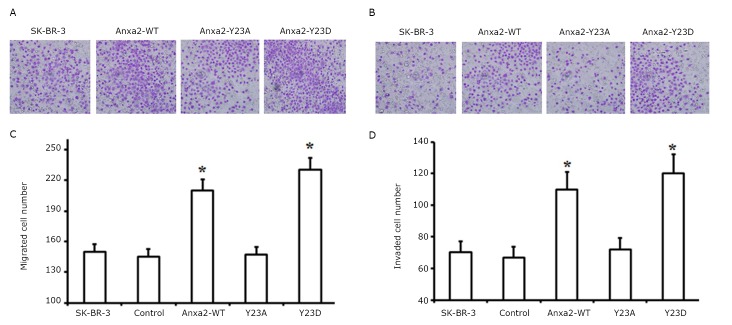
Anxa2-WT and Anxa2-Y23D increased the migration and invasion of SK-BR-3 cells *in vitro*. A and C: Migration assay revealed that the numbers of Anxa2-WT and Anxa2-Y23D cells attached to the bottom of the membrane were notably higher than those of the control cells. B and D: Invasion assay showed the same trend as the migration assay. Each column and bar shows the mean ± SD. Triplicate experiments were done for A and B. The statistical significance was assessed by one-way ANOVA. * *P*<0.05 *vs.* control.

A Boyden chamber assay showed that Anxa2-WT and Anxa2-Y23D increased both the migration and invasion of SK-BR-3 cells (**Figure 3**). In contrast, Anxa2-Y23A had no effect on the migration and invasion of SK-BR-3 cells because of its similar ability to promote migration and invasion to those of the SK-BR-3 and control cells.

### Co-immunoprecipitation of Anxa2 and Stat3 in SK-BR-3 cells

**Figure 4:**
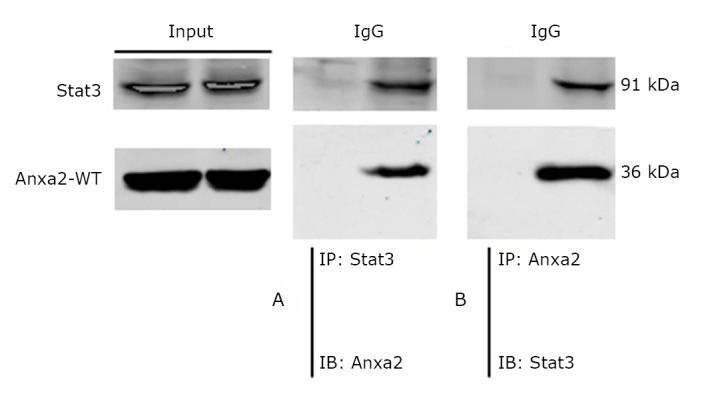
Co-immunoprecipitation of Anxa2 and Stat3 in SK-BR-3 cells. Immunoprecipitation (IP) and immunoblotting (IB). A and B: The SK-BR-3 cell lysate was immunoprecipitated with the indicated antibodies and immunoblotted with the corresponding antibodies.

Co-immunoprecipitation was carried out to study the interaction between Anxa2 and the signal transducer and activator of transcription-3 (Stat3). The SK-BR-3 whole-cell lysates, and protein G-Sepharose beads, and antibodies against Anxa2 and Stat3 were employed to carry out the experiment. **Figure 4** shows that the Anxa2 protein was pulled down by the anti-Stat3 antibody, and Stat3 was co-immunoprecipitated with Anxa2 by an anti-Anxa2 antibody. Thus, Anxa2 and Stat3 interacted with each other.

### Anxa2-Y23D increased the expression of Stat3 phosphorylation in nucleus

**Figure 5:**
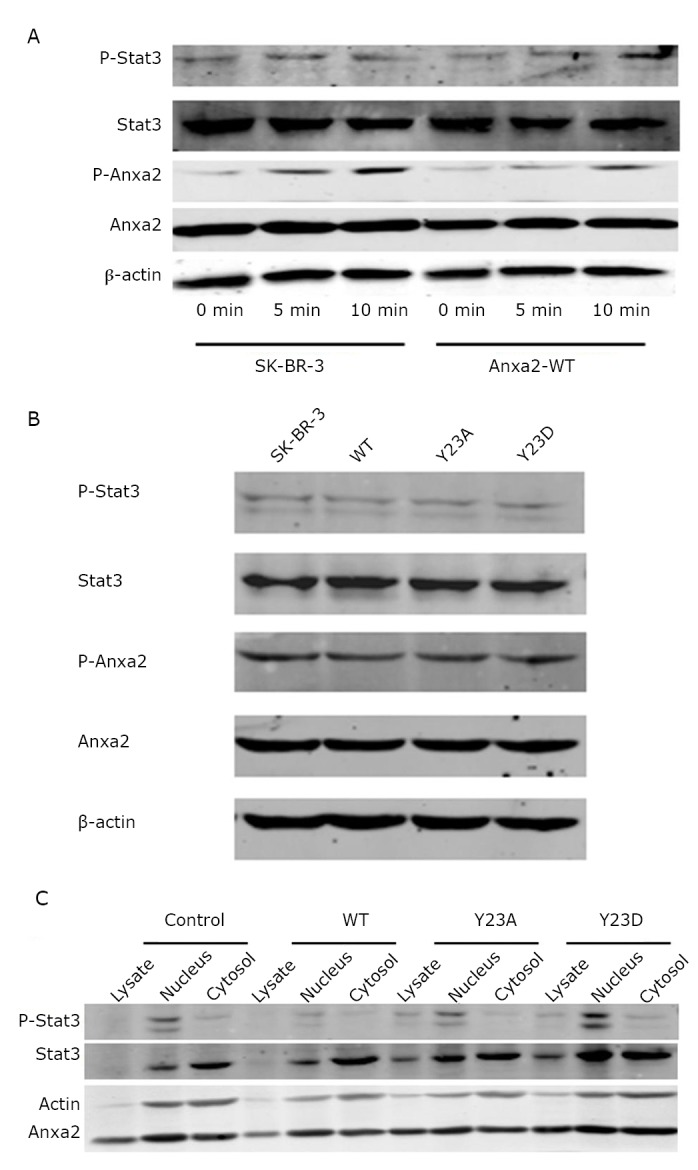
Anxa2-Y23D increased the expression of phosphorylation Stat3 in nucleus. A: Phosphorylation Anxa2 expression increased as the exposure time was lengthened, whereas the expression of other proteins did not show any significant changes. B: Western blot analysis of the expression of Stat3 and phosphorylation Stat3, Anxa2 and Phosphorylation Anxa2 in SK-BR-3, Anxa2-WT, Anxa2-Y23A and Anxa2-Y23D cells, with no significant differences. C: Nucleoprotein and cytosolic protein were extracted from SK-BR-3, Anxa2-WT, Anxa2-Y23A, and Anxa2-Y23D cells. Western blot analysis showed that the expression of phosphorylation Stat3 was notably enhanced in Anxa2-Y23D cells.

The interaction between Anxa2 and Stat3 in the SK-BR-3 cells *in vitro* led to the hypothesis that Stat3 and the phosphorylation Stat3 (P-Stat3) may be up-regulated in Anxa2-WT or Anxa2-Y23D cells. Western blot results suggested no significant differences in the expression of Stat3 and phosphorylation Stat3 (P-Stat3) in both SK-BR-3 and Anxa2-WT cells among different groups exposed to EGF for 0, 5, and 10 min (**Figure 5A**). However, the expression of phosphorylation Anxa2 (P-Anxa2) was increased when the cells were stimulated by EGF for 10 min (**Figure 5A**). The expression of Stat3 and phosphorylation Stat3, Anxa2 and phosphorylation Anxa2 also exhibited no significant differences among the SK-BR-3, Anxa2-WT, Anxa2-Y23A, and Anxa2-Y23D cells (**Figure 5B**). Anxa3-WT and Anxa2-Y23D clearly had no effect on the expression of Stat3 and phosphorylation Stat3 in the total cell lysate. A panel of nucleoproteins and cytosolic protein were extracted from SK-BR-3, Anxa2-WT, Anxa2-Y23A, and Anxa2-Y23D cells, and Western blot analysis showed that phosphorylation Stat3 was notably localized in the nucleus of Anxa2-Y23D cells (**Figure 5C**).

## Discussion

Anxa2 is up-regulated in various tumor types and plays multiple roles in cellular functions, including tumorigenesis, proliferation, cell migration, invasion and adhesion^[^^3^^,^^8^^-^^13^^]^. A previously published study links the down-regulation of Anxa2 with decreasing MCF-7/ADR cell proliferation and invasion^[^^14^^]^. By contrast, the up-regulation of Anxa2 promotes the proliferation and invasion of breast cancer MCF-7 cells^[^^15^^]^. Anxa2 belongs to the Annexin family of calcium binding proteins^[^^16^^]^. Anxa2 has a C-terminal domain with binding sites for calcium and a functional N-terminal domain with p11 protein and t-PA binding sites. Anxa2 N-terminus also bears two phosphorylation sites, on Tyr23 and Ser25, which are presumably targets for Src kinase and protein kinase C, respectively^[^^17^^]^. The phosphorylation of Ser25 may play a role in granule aggregation. The phosphorylation of Tyr23 by Src kinase was proposed to act either as a targeting signal for the plasma membrane together with p11 or as a negative signal for (Anxa2)_2_-(p11)_2_ heterotetramer formation or stabilization^[^^18^^,^^19^^]^. Anxa2 can be phosphorylated on Tyr23 by Src kinase both *in vivo* and *in vitro*^[^^20^^-^^23^^]^. And constitutively phosphorylated with Tyr23 leads to the cell-surface localization of Anxa2, which is essential for the invasion and metastases of pancreatic cancer^[^^2^^]^. Moreover, Src kinase-mediated phosphorylation of Anxa2 on 23Tyr regulates the cytoskeletal dynamics, such as cell scattering and the epithelial mesenchymal transition (EMT)^[^^4^^]^.

In this study, we establish three new findings that elucidate the role of Anxa2 in the proliferation and invasion of breast cancer SK-BR-3 cells, and the effect of Anxa2 on the stimulation of Stat3 pathways. First, the Tyr23 phosphorylation of Anxa2 contributes to the *in vitro* proliferation and invasion of SK-BR-3 cells. We generated a panel of lentivirus plasmids expressing Anxa2-WT, Anxa2-Y23A in which Tyr23 was altered into an alanine residue to create non-phosphorylatable mutant, or Anxa2-Y23D in which Tyr23 was altered into an aspartic acid residue to mimick constitutive phosphorylation. Since Anxa2 is overexpressed in invasive tumors and plays an important role in cancer progression and invasion^[^^12^^]^. Constitutive phosphorylation of Anxa2 also enhances the proliferation, migration and invasion abilities of SK-BR-3 cells.

Second, Anxa2 and Stat3 (signal transduction and activator of transcription3) interact with each other, and have a colocalization in the nucleus. EGF (epidermal growth factor) binding to its receptor (EGFR) induces dimerization and autophosphorylation of the receptor at multiple tyrosine residues, which serve as docking sites for recruitment of proteins with SH2 (Src homology 2) domains that activate multiple downstream signaling pathways. The adaptor protein Grb2 (growth factor receptor-binding protein 2) binds to EGFR, which leads to activation of Stat3 pathways^[^^24^^]^. The tyrosine phosphorylation protein Anxa2 can also been activated by Src-kinase pathways^[^^4^^]^. In the present study, we find that Anxa2 and Stat3 interact with each other, and they have a colocalization in the nucleus of SK-BR-3 cells.

Third, Tyr23 phosphorylation enhances Stat3 phosphorylation and localization in the nucleus. The proline-rich tyrosine kinase (pyk2) along with c-Src, facilitates epidermal growth factor (EGF)-mediated Stat3 reporter gene activation and Stat3 phosphorylation on amino acid residues Tyr-705 and Ser-727^[^^25^^]^. Here, we provide evidence that the expression of phosphorylation Stat3 does not change when the exposure time of the SK-BR-3 cells to EGF are prolonged, or the Anxa2 is up-regulated or constitutively mimicking phosphorylation. However, the phosphorylation Anxa2 is increased with prolonged exposure to EGF, and Stat3 is up-regulated in nucleus when Anxa2 constitutively phosphorylated on Tyr23.

The epidermal growth factor (EGF) and Src kinase induced Stat3 activation mediate series proteins including cell-cycle regulators such as FBJ murine osteosarcoma viral oncogene homolog, mitogen-activated protein kinase 5, c-Myc, and inducers of tumor angiogenesis in invasive breast cancer tissue such as vascular endothelial growth factor, cyclooxygenase-2, matrix metalloproteinase (MMP)-2, MMP-10, and MMP-1^[^^26^^]^. The activation of these proteins trigger proliferation, invasion, migration and oncogenesis. Hepatocyte growth factor (HGF) induces Src kinase activation phosphorylates Anxa2 on Tyr23, and further mediates cell scattering, branching, and the cytoskeletal dynamics, which are necessary for the epithelial mesenchymal transition (EMT). Both Anxa2 and Stat3 can be activated by Src kinase pathway. The present study finds an interaction between Anxa2 and Stat3, and phosphorylation Anxa2 on Tyr23 enhances Stat3 phosphorylation and localization in nucleus. We are reasoned to believe that Anxa2, especially phosphorylation Anxa2 participates in the EGF and Src kinase induced Stat3 activation and Stat3 triggered oncogenesis. The exact machanism of the interaction between Anxa2 and Stat3 will be our future research objective.

Overall, these findings identify the relationship between phosphorylated Anxa2 and phosphorylated Stat3 in the nucleus. Tyr23 phosphorylation of Anxa2 could activate Stat3 and phosphorylation Stat3 localization in the nucleus of breast cancer SK-BR-3 cells.
